# Myeloid Suppressor Cell Depletion Augments Antitumor Activity in Lung Cancer

**DOI:** 10.1371/journal.pone.0040677

**Published:** 2012-07-16

**Authors:** Minu K. Srivastava, Li Zhu, Marni Harris-White, Upendra Kar, Min Huang, Ming F. Johnson, Jay M. Lee, David Elashoff, Robert Strieter, Steven Dubinett, Sherven Sharma

**Affiliations:** 1 Department of Medicine, UCLA Lung Cancer Research Program, David Geffen School of Medicine at UCLA, Los Angeles, California, United States of America; 2 Jonsson Comprehensive Cancer Center, David Geffen School of Medicine at UCLA, Los Angeles, California, United States of America; 3 Department of Biological Chemistry, David Geffen School of Medicine at UCLA, Los Angeles, California, United States of America; 4 Molecular Gene Medicine Laboratory, Veterans Affairs Greater Los Angeles Healthcare System, Los Angeles, California, United States of America; 5 Department of Medicine, University of Virginia, Charlottesville, Virginia, United States of America; Virginia Commonwealth University, United States of America

## Abstract

**Background:**

Myeloid derived suppressor cells (MDSC) are important regulators of immune responses. We evaluated the mechanistic role of MDSC depletion on antigen presenting cell (APC), NK, T cell activities and therapeutic vaccination responses in murine models of lung cancer.

**Principal Findings:**

Individual antibody mediated depletion of MDSC (anti-Gr1 or anti-Ly6G) enhanced the antitumor activity against lung cancer. In comparison to controls, MDSC depletion enhanced the APC activity and increased the frequency and activity of the NK and T cell effectors in the tumor. Compared to controls, the anti-Gr1 or anti-Ly6G treatment led to increased: (i) CD8 T cells, (ii) NK cells, (iii) CD8 T or NK intracytoplasmic expression of IFNγ, perforin and granzyme (iv) CD3 T cells expressing the activation marker CD107a and CXCR3, (v) reduced CD8 T cell IL-10 production in the tumors (vi) reduced tumor angiogenic (VEGF, CXCL2, CXCL5, and Angiopoietin1&2) but enhanced anti-angiogenic (CXCL9 and CXCL10) expression and (vii) reduced tumor staining of endothelial marker Meca 32. Immunocytochemistry of tumor sections showed reduced Gr1 expressing cells with increased CD3 T cell infiltrates in the anti-Gr1 or anti-Ly6G groups. MDSC depletion led to a marked inhibition in tumor growth, enhanced tumor cell apoptosis and reduced migration of the tumors from the primary site to the lung compared to controls. Therapeutic vaccination responses were enhanced *in vivo* following MDSC depletion with 50% of treated mice completely eradicating established tumors. Treated mice that rejected their primary tumors acquired immunological memory against a secondary tumor challenge. The remaining 50% of mice in this group had 20 fold reductions in tumor burden compared to controls.

**Significance:**

Our data demonstrate that targeting MDSC can improve antitumor immune responses suggesting a broad applicability of combined immune based approaches against cancer. This multifaceted approach may prove useful against tumors where MDSC play a role in tumor immune evasion.

## Introduction

Lung cancer remains a daunting health problem with more than 1.1 million deaths attributed to lung cancer worldwide annually [1]. With the existing therapeutic efforts the long term survival for lung cancer patients remains low, thus new therapeutic strategies are needed. Although cancer immunotherapy offers an attractive therapeutic option, activation of the immune system alone is not sufficient for antitumor activity. We anticipate that combined therapies that target pathways of immune activation and mechanisms of immune suppression will be necessary to combat lung cancer. The tumor microenvironment consists of tumor cells, stroma, blood vessels, immune infiltrates and the extracellular matrix. Genetic alterations in oncogenes and tumor suppressor genes or epigenetic changes in the tumor that modulate tumor growth and invasion into the surrounding tissue orchestrate the persistence of inflammatory infiltrates. These cellular infiltrates modulate tumor development and progression. The tumor infiltrates vary by size and composition in diverse tumor types and at different stages of tumor development. The tumor programs the cellular infiltrates to sustain a dysregulated inflammation that is hypo responsive to the tumor. Contributing to the cellular inflammatory infiltrates are myeloid derived suppressor cells (MDSC) that negatively modulate immune responses and promote tumor progression and metastases [2].

MDSC are a heterogeneous population of immature myeloid cells that consists of myeloid progenitors and precursors of macrophages, granulocytes and dendritic cell (DC) [3]. In mice, MDSC are identified by antibodies that detect cell surface expression of Gr1 and CD11b. Increases in the number of MDSC evoke strong natural suppressive activity in cancer patients [4], [5] or tumor-bearing mice [4], [6], [7]. It has been demonstrated that Gr1+CD11b+ immune suppressive cells are capable of inhibiting the T cell proliferative response induced by alloantigens [8], CD3 ligation [9], or various mitogens [10], [11], and can also inhibit IL-2 utilization [12] as well as NK cell activity [5], [13]. These studies indicate that progressive tumor growth is associated with the down-regulation of T cell responses and that the MDSC are involved in negative immunoregulatory mechanisms. In murine tumor models, increases in MDSC in the tumors, spleens, bone marrow and blood downregulates T cell responses [2]. Considering the above information, it is important to understand the impact of MDSC depletion in the modulation of immune responses in lung cancer.

In this study, we evaluated the contribution of the Gr1 or Ly6G expressing myelomonocytic cells on 3LL tumor growth and therapeutic vaccination responses in C57BL/6 mice. Our results demonstrate that MDSC depletion increased APC activity and augmented the frequency and activity of NK and T cells effectors that led to reduced tumor growth, enhanced therapeutic vaccination responses and conferred immunological memory. Our data provides support for the development of agents that target MDSC for combined immune based therapeutic approaches in lung cancer.

## Materials and Methods

### Cell Lines and Reagents

The murine Lewis lung carcinoma (3LL, H-2^b^, also known as LLC, ATCC CRL-1642) obtained from American Type Culture Collection (Manassas, VA), DC2.4 (kind gift from K. L. Rock Dana Farber Cancer Institute, Boston, Mass.) [14] and the β galactosidase reporter T cell hybridoma (B3Z) which recognizes the k^b^ Class I molecule and an ovalbumen (OVA) peptide, SL8 (SIINFEKL) was obtained from N Shastri (UC Berkeley, CA) [15] were used in these studies. 3LL-OVA cells were generated by transfecting 3LL parental cells with the OVA constructs obtained from Dr. Frelinger (University of Rochester, NY). The expression vectors encoding either the full-length Ova or truncated Ova-(138-386) (Ova non-secretory) were transfected using Lipofectin (GIBCO/BRL) according to the manufacturer’s instructions. Selection with the appropriate drug was performed as described [16]. Stable 3LL-OVA transfectants were selected following OVA ELISA of cell lysates and cloned by limited dilution in 96-well plates. For the experiments described in this study we used the 3LL-OVA that expressed the truncated Ova-(138-386). The culture medium (CM) contained RPMI 1640 (Irvine Scientific, Santa Ana, CA) supplemented with 10% fetal bovine serum (Gemini Bioproducts, Calabasas, CA), penicillin (100 units/ml), streptomycin (0.1 mg/ml), and 2 mmol/L glutamine (JRH Biosciences, Lenexa, KS). Fluorescein isothiocyanate-, phycoerythrin-, allophycocyanin-, PerCP- or APC-Cy7-conjugated anti-mouse mAbs to CD3 (145-2C11), CD4 (RM4-5), CD69 (H1.2F3) and CD8a (53-6.7) were purchased from BD Biosciences (San Diego, CA). Fluorescent-conjugated mouse Abs: anti-CD49b, anti-CD11c, anti-CD69, anti-CD44, anti-perforin, anti-granzyme, anti-IL10, anti-IFNγ, anti EpCam and anti-CD107a were from eBioscience (San Diego, CA) and anti-Gr1, anti-CD45 and anti-CD11b were from Biolegend (San Diego, CA). Neutralizing Abs to: Gr1 (RB6-8C5), Ly6G (1A8) were from BioXCell (West Lebanon, NH) and Keratin was from Sigma. IL-2, IFNγ, IL-12, IL-10 and TNFα were quantified with cytokine specific ELISA kits (eBioScience). Sensitivity: IL-2 (3 pg/ml), IFNγ (3 pg/ml), IL-12 (3–5 pg/ml), IL-10 (30 pg/ml) and TNFα (8 pg/ml). Ovalbumen protein and Bradford protein quantification dye was obtained from Sigma (St. Louis, MO). Tissue digestion buffer consisted of [0.2 mg/ml of Collagenase A (Boehringer Mannheim/Roche, Indianapolis, IN), DNAse 25 U/ml (Sigma), and 0.3 U/ml of Dispase (Invitrogen, Carlsbad, CA) in RPMI. T cell purification columns were purchased from R&D (Minneapolis) and MDSC magnetic separation Ab was from Miltenyi Biotech (Auburn, CA). RNA isolation kit was from Qiagen (Valencia, CA) and cDNA kit from BioRad (Hercules, CA) and real time PCR primers were from IDT (Coralville, Iowa). Carboxyfluorescein succinimidyl ester (CFSE) was obtained from Invitrogen.

### Cell Culture

Cells (3LL, DC 2.4, B3Z and 3LL-OVA) were routinely cultured in Corning T75 cm^2^ tissue culture flask in humidified atmosphere containing 5% CO_2_ in air in culture medium (CM). The cell lines were *Mycoplasma* and murine viral pathogen free. The cell lines were used up to the 10^th^ passage. Bone marrow (BM) was harvested by flushing the femurs of C57BL/6 mice with RPMI supplemented with 20% fetal bovine medium (RP-20). The pooled marrow cells were plated in RP-20 supplemented with 1% penicillin-streptomycin and cultured for 72 hrs on flasks coated with 2% gelatin (Sigma, St Louis MO). Non-adherent cells were washed off and adherent cells expanded in RP-20. Following the culture period (11–14 days), single cell suspensions of cultured BMA cells were stained for cell surface markers for: monocytes (CD11b^+^), macrophages (CD11b^+^/F4/80^+^), stromal cells (CD45^−^/CD11b^−^/CD44^+^/CD34^−^), DC (CD11c^+^, DEC205^+^) and B cells (CD19^+^) and evaluated by flow cytometry analyses.

### Tumorigenesis Model

Pathogen- free C57BL/6 mice (6–8wk old; Charles River) were maintained in the West Los Angeles Veterans Affairs Animal Research vivarium in accord with the institution’s animal review board guidelines. All animal work was conducted in accord with the Veterans Affairs Institutional Animal care and Use Committee guidelines: id A3002-01. The Veterans Affairs Institutional Animal care and Use Committee review board approved all the studies involving animals in this manuscript. Animals exhibiting signs of pain or meeting the endpoint criteria were euthanized immediately according to the accepted institution based protocol.

Mice were monitored daily for signs of distress from the tumor burden and to ameliorate pain and suffering mice were euthanized if the animals exhibited any clinical signs of distress, such as loss of appetite, 10% cachexia weight loss, loss of mobility, restlessness, depression, respiratory distress, tumor/skin breakdown, or failure to groom. 3LL tumor cells (2.0×10^5^) were injected *s.c.* in the right supra scapular area of C57BL/6 mice. Tumor volumes were monitored by measuring two bisecting diameters of each tumor with calipers. Tumor volumes were calculated using the formula: V = 0.4ab^2^, (a = large diameter and b = small diameter). We have used two doses for the *in vivo* depletion of MDSC based on previous studies [100 µg/dose [17]] or [200 µg/dose] administered every other day [18], [19]. For studies described in this manuscript we utilized the 200 µg/dose. One week following tumor inoculation, mice with palpable tumors were injected *i.p.* individually with anti-Gr-1-specific (200 µg/dose), or anti-Ly6G specific (200 µg/dose), or isotype IgG2b Ab (200 µg/dose) every 48 hrs for 2 weeks. One, two or three weeks post tumor implantation, tumors, spleen, bone marrow and blood were harvested for evaluation of the frequency and activity of leukocytic populations by specific staining/flow cytometric analyses.

### Orthotopic Model

Implantation of the tumors in the lung was performed as previously described [20]. Briefly, 10^4^ 3LL cells in 25 µl sterile NS diluent were injected by the transthoracic route of C57BL/6 mice utilizing a tuberculin syringe with a 30-gauge needle in the left lung under ketamine/xylazine anesthesia. One week following tumor inoculation, a group of mice were sacrificed to confirm and determine the baseline tumor burden before initiation of therapy. One week following tumor inoculation, mice were individually treated with control isotype or anti-Gr1Ab (200 µg/dose) via *i.p.* route every 48 hrs for 4 weeks. Five weeks following tumor implantation, lungs were perfused and harvested for evaluation of tumor burden by H&E staining of tumor sections. Tumor burden was evaluated in a single cell suspension of lung tumor digests by EpCam staining of tumor cells followed by flow cytometric evaluation. A total of 20,000 events were acquired on the FACSCanto flow cytometer and data analyzed by the FCS Express 3 software.

### Vaccination Model

3LL-OVA tumor cells (2.0×10^5^) were injected *s.c.* in the right supra scapular area of C57BL/6 mice. The vaccine consisted of bone marrow adherent cells (BMA) that had been pulsed with OVA protein. Briefly, BMA cells were pulsed with OVA protein (2.5 mg/ml) in CM at 37°C in an incubator with a humidified atmosphere containing 5% CO_2_ in air for 6 hrs. Cells were washed in PBS twice and resuspended in normal saline (0.1×10^6^ in 200 µl/mouse) and administered by *s.c.* injection on the left contra-lateral flank of the tumor implantation on days 7 and 14 post tumor inoculation. Groups included: (i) Diluent, (ii) BMA-OVA, (iii) BMA-OVA + isotype and (iv) BMA-OVA + anti-Gr1. Seven-days post tumor inoculation, the isotype or anti-Gr1 Ab (200 µg/dose) were administered every 48 hrs for 2 weeks. Tumor burden was monitored as described above and H&E or immunohistochemistry (IHC) staining was performed on the tumor sections. Treated mice that had completely rejected the 3LL-OVA tumors were re-challenged with 3LL-OVA tumor cells (2×10^5^) on the left flank and monitored for tumor growth.

**Figure 1:**
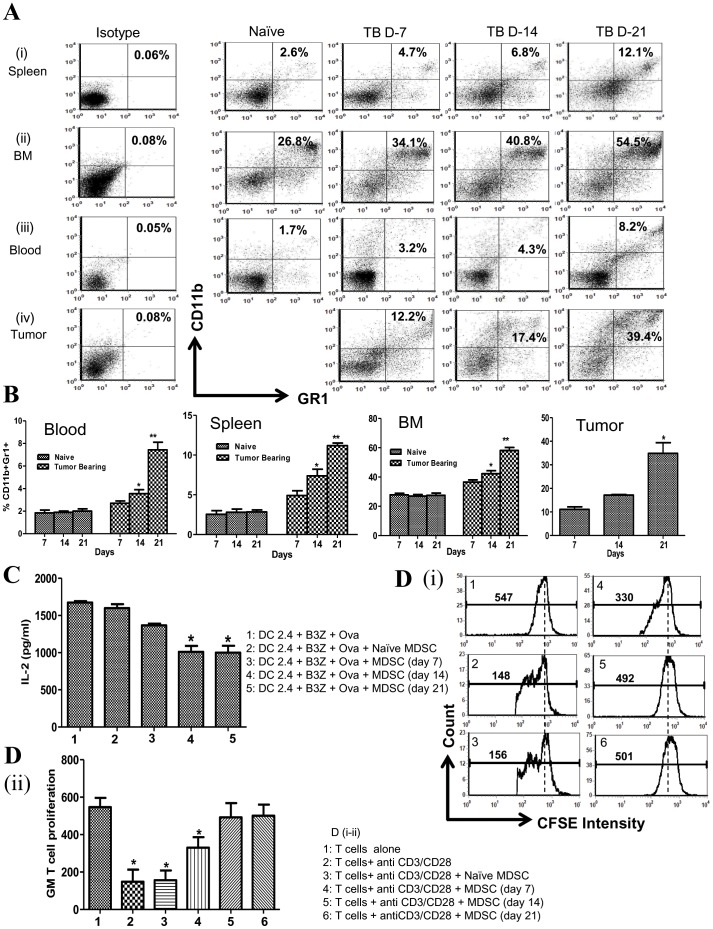
MDSC increased in tumor bearing mice as a function of tumor growth. ***1A*** *,* representative FACS plots for MDSC in blood, BM, spleen and tumor of 3LL tumor bearing mice (Days 7, 14 and 21) in comparison to naive mice. ***1B*** *,* FACS data for MDSC frequency in blood, BM, spleen and tumor (Days 7, 14 and 21) represented as bar graphs (*p<0.05 day 14 vs day 7), (**p<0.005 day 21 vs day 14). ***1C*** *,* MDSC from tumor bearing mice suppress DC2.4 APC activity to activate the antigen specific CD8 T cells to secrete IL-2 (*p<0.05 with MDSC compared to without MDSC), ***1D*** *,* MDSC from tumor bearing mice suppress anti-CD3/CD28 stimulated CFSE labeled T cell proliferation. ***1Di*** *,* Representative FACS CFSE intensity plots for T cell proliferation (with or without MDSC). ***1D-ii*** *,* Geometric mean (GM) for T cells CFSE intensity represented as bar graphs. (*p<0.05 with MDSC vs without MDSC), values reflect mean ± standard error of the mean (SEM), n = 8 mice per group.

### Immunological Memory

Splenocytes from mice that had rejected the secondary tumor challenge were monitored for T cell immune memory phenotype by staining for T cell memory surface markers CD44, CD69 and intracytoplasmic IFNγ with or without stimulation with OVA protein (2.5 mg/ml overnight) *in vitro*. IFNγ secreted by spenocytes (5×10^6^) in the culture supernatant was quantified by ELISA and CD4 and CD8 T cell IFNγ or IL-10 production was quantified by staining/flow cytometry. Splenocytes from naïve mice served as controls.

**Figure 2:**
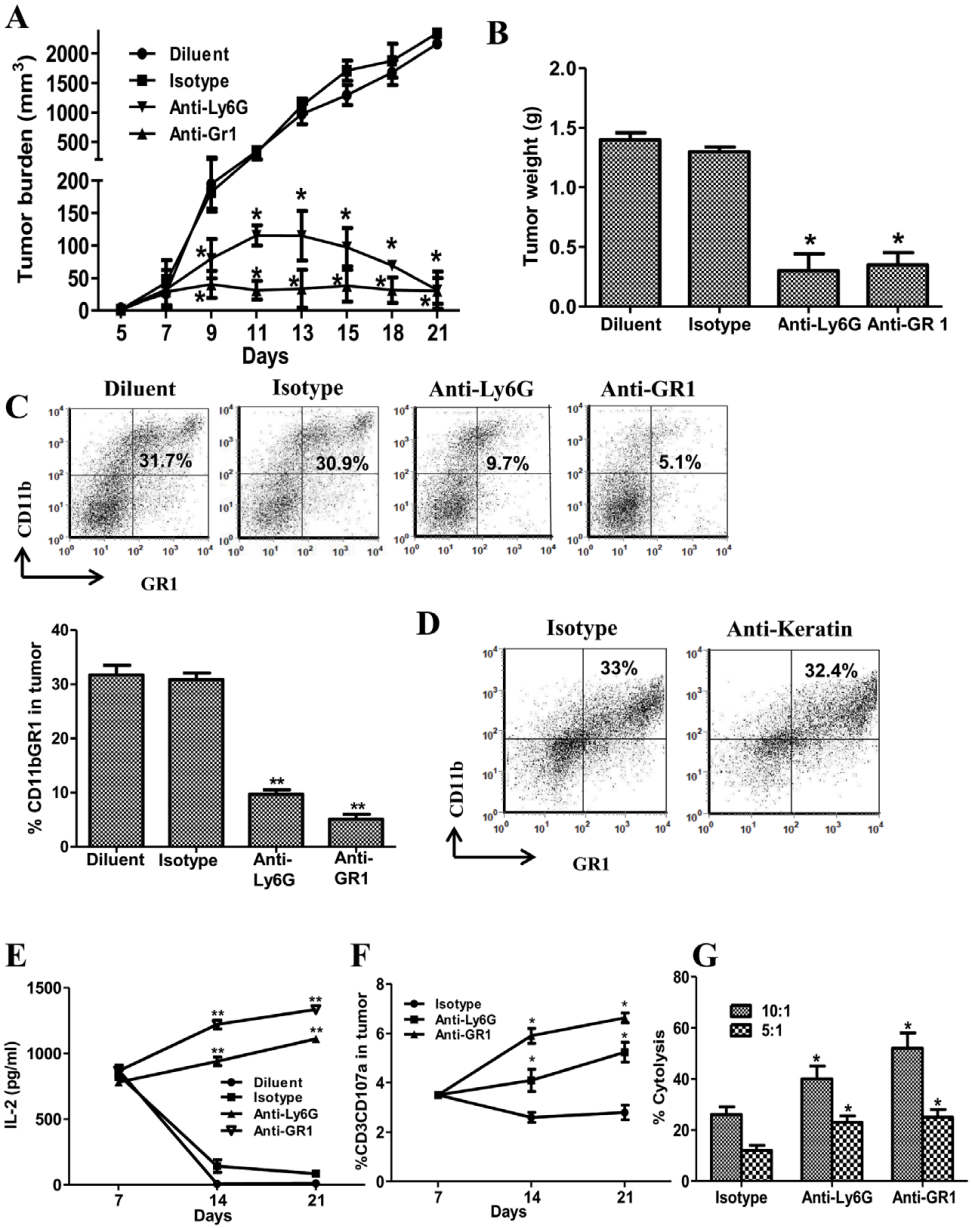
MDSC depletion reduced 3LL tumor burden, increased APC activity and augmented splenic T cell cytolytic function. ***2A*** *,* Changes in tumor volume following anti-Gr1 or anti-Ly6G Ab mediated MDSC depletion. ***2B*** *,* Changes in tumor weights for MDSC depleted and control groups (day 21). ***2C*** *,* Representative FACS plots and bar graphs for frequency of CD11b+Gr1+ MDSC in tumor following anti-Gr1 or anti-Ly6G Ab treatment. ***2D*** *,* Representative FACS plots for frequency of CD11b+Gr1+ MDSC in tumor following anti-keratin Ab treatment. ***2E*** *,* Representative APC activity in tumor to activate the CD8 antigen specific T cells to secrete IL-2 following anti-Gr1, anti-Ly6G, isotype control Ab and diluent treatment. ***2F*** *,* CD107a activation marker expression on T cells in tumor following MDSC depletion. ***2G*** *,* Total purified splenic T cell cytolysis against 3LL parental tumor (E:T 10:1, 5:1) for the MDSC depleted groups and isotype controls. There were no differences in T cell cytolysis of parental 3LL cells between the diluent and the isotype control group (data not shown). There were no changes in T cell cytolysis against the non related B16 tumors between the MDSC depleted groups and controls (data not shown). Data in all panels are representative of 2-3 independent experiments (Data, mean ± SEM, p values: compared to controls *p<0.05, **p<0.005, n = 6 mice/group.

### Antigen Processing and Presentation Assay

DC 2.4 or BMA or a single cell suspension of tumor digest (5–10×10^4^ c/well) from controls, anti-Gr1 or anti-Ly6G treated tumor bearing mice were co-cultured with OVA protein (2.5 mg/ml) and the MHC Class I restricted CD8 T cell line B3Z (10^5^ c/well) in CM in triplicate wells of a 96-well plate for 24 hrs. IL-2 secreted by the activated CD8 T cells in the supernatant was quantified by ELISA.

**Figure 3:**
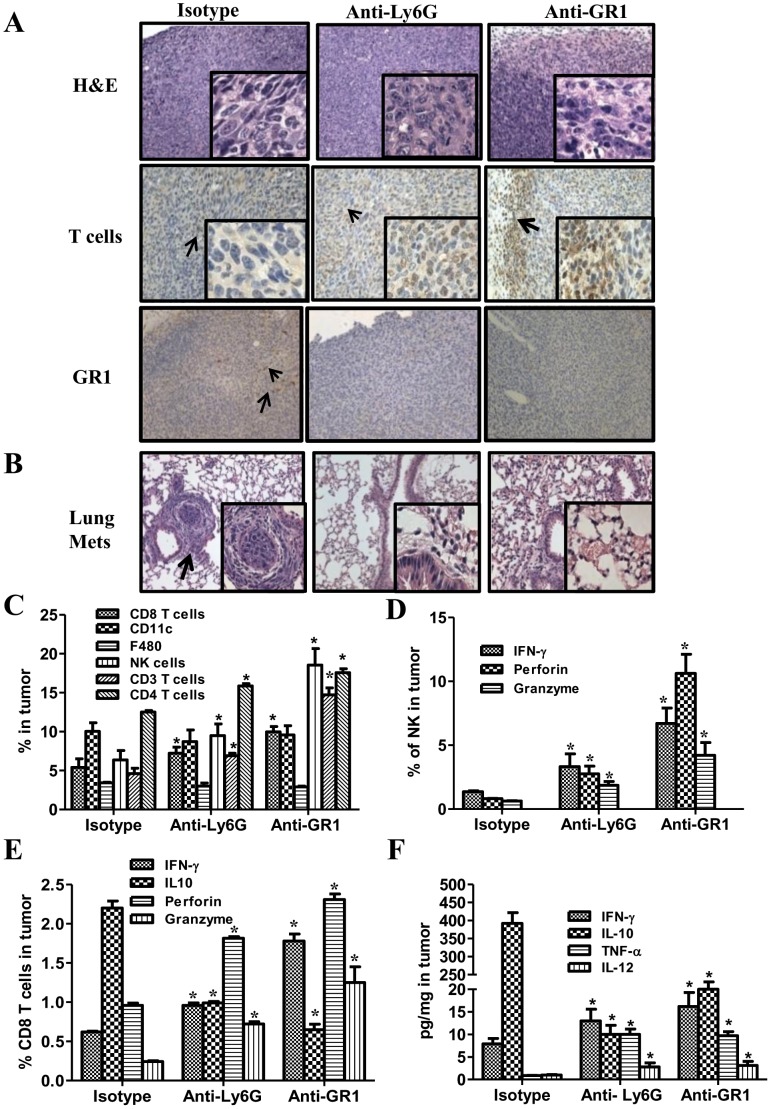
MDSC depletion augmented the frequency and function of NK and T cell effectors and reduced 3LL lung metastases in tumor bearing mice. ***3A*** *,* H&E and IHC for T cells or GR1 expressing cells in tumor tissues following MDSC depletion or control treatments. ***3B*** *,* H&E of lung sections to evaluate lung metastasis following MDSC depletion. ***3C*** *,* Frequency of tumor infiltrates (CD3, CD4, CD8, CD11c, F480 or CD49b), **3** ***D*** *,* NK cells expressing IFNγ, perforin and granzyme, ***3E***, CD8 expressing IFNγ, perforin and granzyme but reduced IL-10 expression analyzed by flow cytometry. ***3F*** *,* Tumor cytokine expression **(**IFNγ, IL-12, TNFα and IL-10) following MDSC depletion. (Data, mean ± SEM, *p<0.05 compared to controls, n = 8 mice/group).

### Flow Cytometry

Flow cytometry was performed for the following T cell surface markers CD3, CD4, CD8, CD69 on single cell suspension of splenocytes or tumor digests following treatment as described above. Splenocytes and tumor cell digests were also evaluated for the NK cells with the surface marker CD49b. CD4 T, CD8 T and NK cells were individually evaluated for intracytoplasmic perforin, granzyme, IFNγ or IL-10. CD107a on T cells were evaluated by cell surface staining/flow cytometry. For analyses of leukocytic infiltrates in the tumor tissue, tumors were mechanically dissociated on a wire mesh by crushing with a 10 ml syringe and incubated in tissue digestion buffer at 37°C for 25 min. The cells were filtered through 70 µm nylon strainers (BD Biosciences, Bedford, MA), stained with specific markers and analyzed by flow cytometry. Samples were acquired on a FACSCanto (BD Biosciences/FACSCalibur flow cytometer (Becton Dickinson, San Jose, CA) in the University of California, Los Angeles, Jonsson Cancer Center Flow Cytometry Core Facility. A total of 10,000 to 25,000 events were acquired and cells staining positive within the total live cell population were analyzed using FCS Express 3 (De Novo Software, Canada). Cells incubated with irrelevant isotype-matched antibodies and unstained cells served as controls.

**Figure 4:**
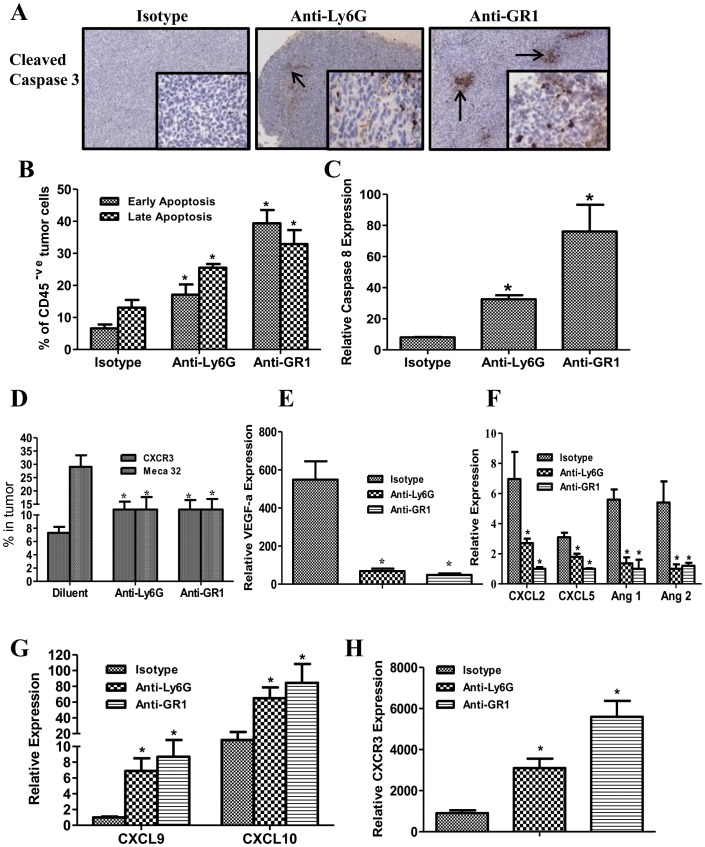
MDSC depletion augmented the tumor cell apoptosis and increased anti-angiogenic but reduced pro-angiogenic marker expression in tumors. ***4A*** *,* IHC for cleaved caspase 3 in tumor sections following MDSC depletion. The arrows are indicative of cleaved caspase 3 staining. ***4B*** *,* AnnexinV and PI staining in tumor digests by flow cytometry. ***4C*** *,* Relative caspase 8 expression by QRTPCR normalized with β-actin expression in tumor tissues. ***4D*** *,* Endothelial MECA 32 cell marker expression and total T cell CXCR3 expression in tumors by flow cytometry. ***4E–H*** *,* Relative pro-angiogenic and anti-angiogenic marker expression in tumors by QRTPCR normalized by β-actin. MDSC depletion decreased pro-angiogenic (VEGF-a, CXCL2, CXCL5, Ang1 and Ang2) (**4E–F**) but increased anti-angiogenic (CXCL9 and CXCL10) (**4G**) and CXCR3 (**4H**) expression in the tumors. (Data, mean ± SEM, p values: compared to controls *p<0.05, n = 8 mice/group).

**Figure 5:**
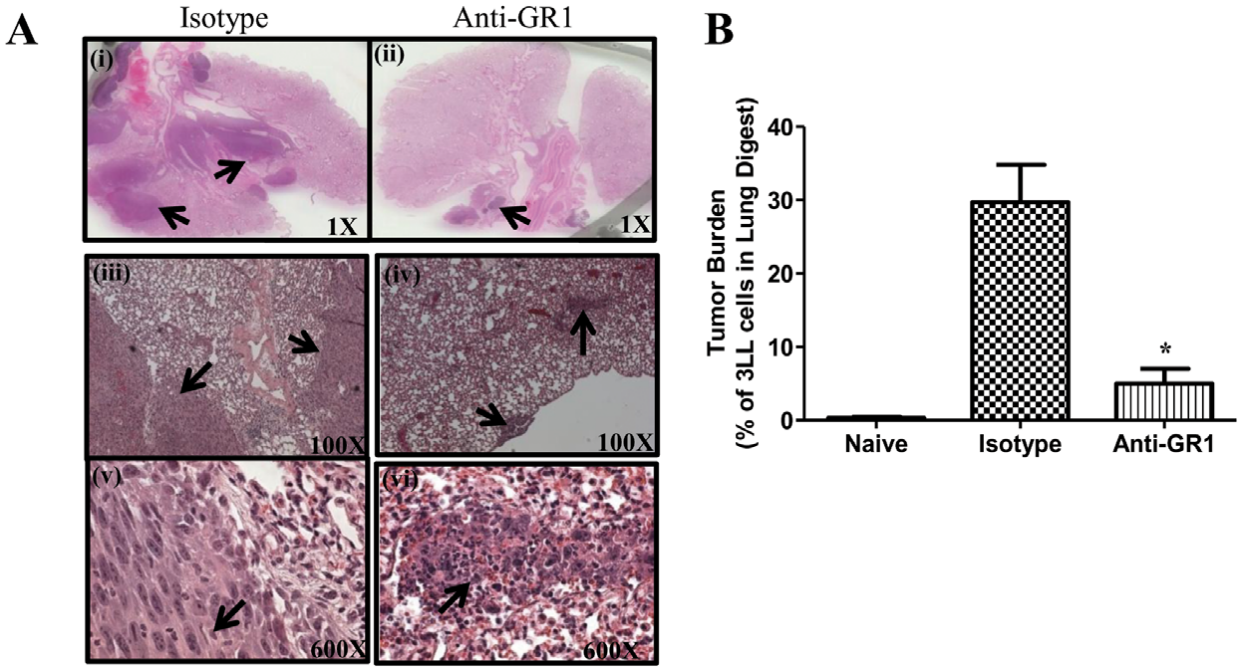
MDSC depletion inhibited tumor burden in the 3LL orthotopic lung tumor model. ***5A*** *,* Representative H&E of lung tumor sections following MDSC depletion and controls; arrows indicative of tumor. ***5B*** *,* Tumor burden evaluated by cell surface staining for Epcam in a single cell suspension of tumor digests and quantified by flow cytometry. (Data, mean ± SEM, p values: compared to controls *p<0.05, n = 8 mice/group).

### CFSE Based Cytolysis Assay

Total cytolytic T cell activity in the spleen against parental 3LL tumor cells or syngeneic B16 melanoma cells were evaluated following treatment with anti-Gr1 or anti-Ly6G Ab on day 21 post tumor inoculation. Tumor targets were labeled with CFSE at dose (1 µM) in PBS for 15–20 mins according to the manufacturer’s instructions. After washing, the labeled targets were incubated with T cells purified from spleens using T cell columns (R&D), and cytolytic activities were evaluated against autologous 3LL tumor cell line and the syngeneic control B16 melanoma tumor cell line. The purified splenic T cell effectors were co-cultured with tumor cell targets (E:T of 10∶1–5∶1) for four hours in quadruplet wells in a 96-well plate. Following a co-culture, cells were washed and analyzed by flow cytometry. Decreases in the frequency and intensity of CFSE labeled cells were used to calculate % of cytolysis in tumor targets.

**Figure 6:**
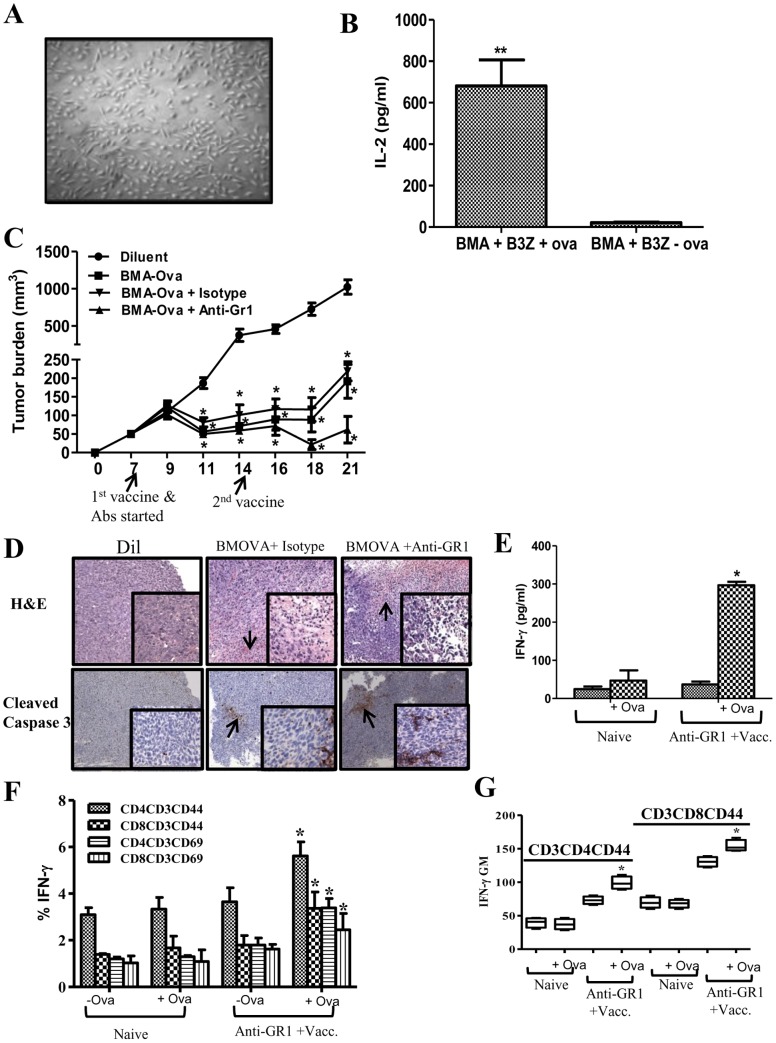
MDSC depletion enhanced vaccination responses. ***6A*** *,* 14-day BMA culture propagated in CM. ***6B*** *,* BMA cells processed and presented the OVA protein to activate the CD8 T OVA specific cell line to secrete IL-2. ***6C*** *,* Tumor volume following vaccination (groups: Diluent, BMA-OVA, BMA-OVA + Isotype and BMA-OVA + anti-Gr1 Ab). ***6D*** *,* H&E and IHC for cleaved caspase 3 in tumor tissues following vaccination. Arrows indicative of leukocytic infiltrates and cleaved caspase 3 staining in the tumors. ***6E–G*** *,* Flow cytometry analyses of splenic T cell memory and activation markers from mice that had rejected tumors in the vaccination plus anti-Gr1 treatment group in comparison to non tumor bearing naïve controls; **6E**, Splenic production of IFNγ, **6F**, Frequency of IFNγ producing CD4 and CD8 memory (CD44) and activation (CD69) marker expressing T cells. **6G**, Geometric Mean of IFNγ producing memory T cells from vaccination plus anti-Gr1 treatment following stimulation with OVA protein compared to naïve controls. (Data, mean ± SEM, p values: compared to controls *p<0.05, n = 8 mice/group).

**Table 1:** 50% of mice in the BMA-OVA + anti-Gr1 Ab treatment group completely rejected their primary tumors and 3LL-OVA tumor cells (2×10^5^) on re-challenge.

Groups	Regression
Diluent	0/8
BMA-Ova	0/8
BMA-Ova + Isotype	0/8
BMA-Ova + Anti-GR1	4/8

### CFSE Labeling and T Cell Suppression Assays

T cells were purified from spleens using the T cell isolation columns. Following two washes with PBS, purified T cells were (2×10^6^ cells/ml) mixed with an equal volume of 10 µM CFSE solution for 10 min. The reaction was terminated by the addition of 10 volumes of cold RPMI 1640/10% FBS. Labeled cells (1×10^6^ cells/ml) were washed twice with PBS/2% FBS. In a U-bottom 96-well cell culture plate pre-coated with anti-CD3 Ab (5 µg/ml), purified T cells (2×10^5^) were cultured in DMEM supplemented with 10% FBS and anti-CD28 Ab (5 µg/ml) for 72–96 h. To determine the impact of MDSC on T cell proliferation, CFSE labeled T cells were co-cultured with purified Gr1 splenic MDSC (2–4×10^5^) from 3LL bearing mice or naïve mice and changes in proliferation was assessed by flow cytometry. MDSC were isolated from spleens by labeling the cell suspensions with rat anti mouse Gr1 mAb (eBioscience), followed by magnetic antibody cell separation using anti-rat microbeads (Miltenyi Biotec). The isolated cells were >90% pure for CD11b+Gr1+ expressing MDSC as determined by flow cytometry.

### Cytokine ELISA

The cytokines (IFNγ, TNFα, IL-10 and IL-12) in splenocyte culture supernatants or from spleen or tumor homogenates were determined by ELISA and the plates read at the specified wavelengths with a Microplate Reader (Amersham Biosciences, Sunnyvale, CA).

### Tumor Tissue Sectioning and Immunohistochemistry

To determine the extent of lymphocytes infiltrating the tumors from the various treatment groups, C57BL/6 mice bearing 7-day tumors were injected *i.p.* with Gr1-specific (200 µg/dose) or Ly6G specific (200 µg/dose) or isotype IgG2b Ab (200 µg/dose) every 48 hrs for 2 weeks. Non-necrotic tumors were isolated and embedded in paraffin and serially sectioned to 5-µm thickness. Sections were H&E or immune stained for CD3 T lymphocytes or Gr1 expressing cells. To determine apoptotic tumor cells, tumor sections were stained for cleaved caspase 3. Antigen retrieval was accomplished with sodium citrate (10 mmol/L, pH 6.0). Sections were blocked with 10% normal goat serum, and probed with an antibody against CD3, Gr1 or cleaved caspase 3. Primary antibodies were incubated overnight at 4°C. After incubation with secondary antibody (Vector Laboratories), staining was developed using DAB substrate kit for Peroxidase (SK-4100, Vector Laboratories). Counter-stain was achieved with hematoxylin. The slides were observed under 1X71 Olympus Fluorescence microscope attached to a CCD camera. The images were acquired with 10X, 20X and 60X objectives using the Image Pro software.

### Apoptosis

To determine the extent of tumor cell apoptosis, a single cell suspension of the tumor tissues were stained using a PI/Annexin V-FITC apoptosis detection kit (BD Pharmingen) according to the manufacturer’s instruction and the percentage of apoptotic tumor cells (gated on CD45^−^ cells) were analyzed by flow cytometry.

### Total RNA Preparation, cDNA Synthesis and Real-time QPCR

Mice bearing seven-day old tumors were treated with isotype, anti-Gr1 or anti-Ly6G Ab and 2 week following treatment, tumor tissues were quantified for *Caspase 8, Angiopoietin1, Angiopoietin2, VEGF-a, CXCL2, CXCL5, CXCL9, CXCL10 and CXCR3* gene expression using a SYBR Green Quantitative PCR Kit in the iCycler (Bio-Rad) and corrected with the β-*actin* housekeeping control gene. For QPCR analyses, RNA was isolated using a Qiagen kit. The cDNA was prepared with a kit (BioRad) according to the manufacturer’s instructions. Amplifications were done in a total volume of 25 µl for 40 cycles of 15 s at 95°C, 20 s at 60°C, and 30 s at 72°C. Primer sequences were as follows: β-*actin* F, 5′- CCACAGCTGAGAGGGAAATC -3′ and R, 5′- TCTCCAGGGAGGAAGAGGAT -3′; *Caspase* 8 F, 5′- TGCTTGGACTACATCCCACAC-3′ and R, 5′- GTTGCAGTCTAGGAAGTTGACC -3′; *Ang-1* F, 5′- TCTCATGCTAACAGGAGGTTGGTG -3′ R, 5′-GGATCATCATGGTGGTGGAACGTA-3′; *Ang-2* F, 5′- CAAGAGCTCGGTTGCTATCCGTAA-3′ R, 5′ GTCCATGTCACAGTAGGCCTTGAT3′; *VEGF-a* F, 5′- TGTACCTCCACCATGCCAAGT-3′ R, 5′- CGCTGGTAGACGTCCATGAA-3′; *CXCL5* F, 5′- GGTCCACAGTGCCCTACG-3′ R, 5′- GCGAGTGCATTCCGCTTA-3′; *CXCL2* F, 5′- AGTGAACTGCGCTGTCAATG -3′ R, 5′- GAGAGTGGCTATGACTTCTGTCTG-3′; *CXCL9* F, 5′- GCACGATCCACTACAAATCCC-3′ R, 5′- GGTTTGATCTCCGTTCTTCAGT-3′; *CXCL10* F, 5′- CCAAGTGCTGCCGTCATTTTC-3′ R, 5′- TCCCTATGGCCCTCATTCTCA-3′; and *CXCR3* F, TCTCCCTACGATTATGGGGAAAA-3′ and R, 5′- GGTTCTGTCAAAGTTCAGGCT-3′.

### Statistical Analyses

All data are presented as mean ± SE. Statistical analysis was performed using Prism (GraphPad Software). We used analysis of variance for data with multiple groups, unpaired Student’s *t*-test for dual comparison. *P* values <0.05 were considered significant.

## Results

### MDSC Increased in Tumor Bearing Mice as a Function of Tumor Growth

To determine the impact of 3LL tumor growth on the frequency of MDSC, non necrotic tumors, blood, spleen or BM were evaluated for Gr1+CD11b+ expression (days 7, 14 and 21 post tumor inoculation). Similar to findings of MDSC increase in cancer patients [4], [5], there were increased MDSC frequency in 3LL tumor bearing mice. MDSC frequency increased in the blood (2–4 fold), spleen (2.6–4 fold) and BM (1.5–2 fold) of tumor bearing mice in comparison to control naive mice **(** **Figure 1A–B** **)**. Day-7 tumors had a lower frequency of MDSC in comparison to days 14 and 21. The frequency of MDSC in the tumor digests was further enhanced on day 21 (3 fold) compared to day 14 tumors **(** **Figure 1A–B** **)**. To confirm the immune suppressive effects of MDSC, we evaluated the function of splenic MDSC from tumor bearing mice on the inhibition of DC2.4 APC activity or T cell proliferation. For the *in vitro* DC APC activity evaluation, we selected cell numbers of DC and OVA specific CD8 T cells that produced high levels of IL-2 under the assay conditions to provide clear differences in IL-2 levels in the presence or absence of MDSC. MDSC from naïve mice did not suppress DC APC activity. Although MDSC from day 7 tumor bearing mice suppressed DC APC activity, the suppressive effect was lower in comparison to MDSC from day 14 and 21 tumor bearing mice **(** **Figure 1C** **)**. In addition, MDSC from naïve mice did not inhibit anti-CD3/CD28 stimulated CFSE labeled T cell proliferation and MDSC from day 7 tumor bearing mice had a lower suppressive capacity on T cell proliferation in comparison to MDSC from day 14 and 21 tumor bearing mice **[** **Figure 1D** **(i–ii)]**.

### MDSC Depletion Reduced 3LL Tumor Burden, Increased APC Activity and Augmented the Cytolytic Function of T Cell Effectors

To determine the impact of MDSC on tumor burden, APC and T cell effectors, we depleted MDSC in 3LL tumor bearing mice utilizing anti-Gr1 or anti-Ly6G Ab. In comparison to controls, MDSC depletion led to the inhibition of tumor volume **(** **Figure 2A** **)**, tumor weights (8 fold-day 21) **(** **Figure 2B** **)** and a reduction in Gr1 expressing cells (4–5 fold) in the tumor **(** **Figure 2C** **)** and systemically in the blood (2 fold), spleen (2.4–7 fold) and BM (1.3–2.9 fold) (data not shown). To evaluate the specificity of antibody mediated MDSC depletion, a negative control anti-keratin Ab was administered on the same dose and schedule as the MDSC depletion antibodies. The anti-keratin Ab treatment did not impact the Gr1 expressing MDSC frequency **(** **Figure 2D** **)**. MDSC depletion increased APC activity in the tumor whereas the controls had reduced APC activity on days 14 and 21 in comparison to day 7 **(** **Figure 2E** **)**. Compared to controls, MDSC depletion led to an increase in the frequency of activated CD3 CD107a T effector cells **(** **Figure 2F** **)** and systemic splenic T cell specific cytolysis against CFSE labeled parental tumor [E:T at 10∶1, (1.5–2 fold) and at 5∶1, (2 fold)] **(** **Figure 2G** **)**. There were no changes in T cell cytolysis against the non related B16 tumors between the MDSC depleted groups and controls (data not shown).

### MDSC Depletion Augmented the Frequency and Function of NK and T Cell Effectors and Reduced 3LL Lung Metastases

NK and T cell effectors are decreased in the tumor bearing state. We determined the impact of MDSC depletion on these effectors in 3LL tumor bearing mice. In comparison to controls, MDSC depletion led to small diffused tumors by H&E staining with increased T cell infiltrates and reduced Gr1 expressing cells **(** **Figure 3A** **)**. While the mice in the control groups had visible lung tumor metastases, MDSC depleted groups did not show any visible lung metastases **(** **Figure 3B** **)**. MDSC depletion increased the frequency of NK (1.6–3 fold), CD3 (1.5–3.3 fold), CD4 (1.5 fold), CD8 (1.5–2 fold) but did not alter the frequency of F480^+^ macrophages or CD11c^+^ DC **(** **Figure 3C** **)**. There were no changes in FoxP3+CD4CD25+ T cells in the tumor site and periphery following MDSC depletion with anti-Gr1 or anti Ly6G treatment (data not shown). In comparison to controls, MDSC depletion increased the frequency of activated: (i) NK cells expressing IFNγ (4–6 fold), perforin (3–10 fold) and granzyme (2–4 fold) **(** **Figure 3D** **)** and (ii) CD8 expressing IFNγ (1.5–3 fold), perforin (2–2.5 fold) and granzyme (2.5–6 fold) but reduced IL-10 (2.4–3.6 fold) expression **(** **Figure 3E** **).** There were increased IFNγ (1.6–2 fold), IL-12 (3 fold), and TNFα (10 fold) but reduced IL-10 (19–39 fold) in the tumors following MDSC depletion in comparison to controls **(** **Figure 3F** **)**.

### MDSC Depletion Augmented Tumor Cell Apoptosis, Increased Anti-angiogenic but Reduced Pro-angiogenic Marker Expression in Tumors

Based on the increased frequency and activation profile of NK and T cells effectors in the tumor following MDSC depletion, we evaluated apoptosis of tumor cells by quantifying the expression of cleaved caspase 3 by IHC in the tumor sections and the % of annexin V/PI +ve stained cells on the non CD45 expressing tumor cell population by flow cytometric analysis. IHC of the tumor sections revealed increased tumor staining of cleaved caspase 3 **(** **Figure 4A** **)** and increased % of annexin V/PI +ve apoptotic tumor cells (early: 2.5–5.9 fold, late: 1.9–2.4 fold) **(** **Figure 4B** **)** in the MDSC depleted groups compared to controls. Similarly the tumors from the MDSC depleted group had: enhanced caspase 8 expression (6.4–15 fold) **(** **Figure 4C** **)**, decreased MECA 32 endothelial cell marker expression (2 fold) and increased T cell CXCR3 expression (1.5–2 fold) by flow cytometry **(** **Figure 4D** **)**, decreased pro-angiogenic (VEGF-a, CXCL2, CXCL5, Ang1 and Ang2) **(** **Figure 4** **E–F)** but increased anti-angiogenic (CXCL9 and CXCL10) **(** **Figure 4G** **)** and CXCR3 **(** **Figure 4H** **)** expression in the tumors by RTQPCR. The antitumor efficacy of MDSC depletion with anti-Gr1 antibody was determined in the 7-day established orthotopic 3LL lung cancer model. Similar to the findings in the *s.c.* model, MDSC depletion inhibited tumor burden by 7-fold compared to controls **(** **Figure 5A–B** **)**. In the control groups, there were 7–9% decrease in the average body weight at the end of the experimental duration but no significant weight change was observed in the anti-Gr1 treatment group. Similar to what was observed in the *s.c* model; we found increased NK and T cell effectors in the lung following treatment (data not shown).

### MDSC Depletion Enhanced Therapeutic Vaccination Responses

The impact of MDSC depletion on therapeutic vaccination responses was evaluated utilizing 3LL OVA tumor cells and BMA cells pulsed with the OVA protein served as the vaccine. 14-day BMA culture **(** **Figure 6A** **)** propagated in RP-20 were evaluated by cell surface staining/flow cytometry analyses. BMA culture is comprised of a heterogeneous population of monocytes (51%-CD11b^+^), macrophages (14%-F480^+^CD11b^+^), DC (10%-CD11c^+^, DEC205^+^), stromal cells (20%-CD45^−^/CD11b^−^/CD44^+^/CD34^−^) and B cells (8%-CD19^+^) and cell surface phenotype (MHC Class I (94%), MHC Class II (53%), CD80 (57%) and CD86 (37%) necessary for APC activity. BMA cells efficiently processed and presented the OVA protein to activate the CD8 T OVA specific cell line to secrete IL-2 **(** **Figure 6B** **)**. Mice bearing 3LL-OVA were vaccinated with: (i) Diluent, (ii) BMA-OVA, (iii) BMA-OVA + Isotype and (iv) BMA-OVA + anti-Gr1 Ab. Compared to controls, therapeutic vaccination with BMA-OVA substantially inhibited tumor growth rate without eradicating the tumors (7 fold) **(** **Figure 6C** **)**. In comparison to controls, the combination of BMA-OVA + anti-Gr1 Ab had the most pronounced tumor growth inhibition (20 fold) **(** **Figure 6C** **)** and H&E section revealed increased leukocytic infiltrates and increased tumor staining of cleaved caspase 3 **(** **Figure 6D** **)**. 50% of mice in the combined BMA-OVA plus anti-Gr1 Ab treatment group completely rejected their primary tumors and a rechallenge dose of 3LL-OVA tumor cells (2×10^5^) **(** **Table 1** **)**. Mice that had rejected a tumor rechallenge were evaluated for T cell memory and T cell activation phenotype following splenic stimulation with OVA protein *in vitro*. In comparison to naïve control, mice that had rejected tumors in the vaccination plus anti-Gr1 treatment had increased: splenic production of IFNγ **(** **Figure 6E** **)** and frequency of IFNγ producing CD4 and CD8 memory (CD44) and activation (CD69) marker expressing T cells **(** **Figure 6F** **)**. Following splenic stimulation with OVA protein, memory T cells had increased production of IFNγ from the vaccination plus anti-Gr1 treatment compared to naïve controls **(** **Figure 6G** **)**. The splenic T cell IL-10 expression pattern following OVA stimulation *in vitro* from mice that had rejected a rechallenge did not change in comparison to T cells from naïve control (data not shown).

## Discussion

With the existing therapeutic efforts, the long term survival for lung cancer patients remains low, thus novel therapeutic strategies are needed. Cancer immunotherapy offers an attractive therapeutic option. The concept of immune approaches against lung cancer remains attractive because although surgery, chemotherapy, and radiotherapy alone or in combination produce response rates, relapse is unavoidable. Thus, strategies that harness the immune system against tumors have the potential for long term cancer free survival. Immune therapy for lung cancer has potential; however, there have been limited Phase III trial-documented improvement in survival [21]. Tumor-induced immune suppression may contribute to the limited efficacy of the approaches. Many tumors, including lung cancer, have the capacity to promote immune tolerance and escape host immune surveillance. Tumors utilize numerous pathways to inhibit immune responses, including the elaboration of immune inhibitory cytokines as well as inducing host cells to release immune inhibitors [22], [23]. In addition to these mechanisms, immune suppression through MDSC has a crucial role in promoting tumor progression. We hypothesized that activating immune cells through therapeutic vaccination with simultaneous disruption of MDSC mediated regulatory mechanisms that limit immune responses will improve the antitumor activity in lung cancer. To test our hypothesis, we evaluated the efficacy of a combined immune based approach consisting of a therapeutic vaccine and MDSC depletion on the impact of immune activation and antitumor activity in an established murine tumor model.

To initiate antigen specific responses *in vivo*, we utilized a cellular vaccine, consisting of BMA cells pulsed with the model OVA antigen expressed by the OVA modified 3LL tumor cells. The BMA cells were pulsed with the OVA antigen to allow for antigen processing and presentation and then injected *s.c.* on the contralateral flank of the established tumor to initiate antigen specific anti tumor immune responses. The BMA cells expressed cell surface phenotype for APC activity and efficiently processed and presented antigens to antigen specific CD8 T cells *in vitro* as well as induced antitumor responses *in vivo*. In contrast, although lung cancer cells express tumor antigens, the limited expression of MHC antigens, defective transporter associated with antigen processing (TAP) and lack of co-stimulatory molecules make them ineffective APCs [24].

To circumvent MDSC mediated immune suppression in the tumor microenvironment, we utilized monoclonal antibodies (anti-Gr1 or anti-Ly6G) to deplete MDSC. We have found that as tumors progress, the frequency and activity of MDSC are enhanced in the tumor microenvironment and systemically. Our results show about 40% of tumor infiltrates are MDSC that have the capacity to turn off the functional antitumor activities of APC, NK and T cell effectors. The tumor cell inoculations in this study did not include matrigel mixed with tumor cells but rather tumor cells in saline. Hence the increase in MDSC in the tumor microenvironment and systemically is due to progressive tumor growth. However injection of matrigel formulations that release MDSC chemotactic factors also have the potential to recruit MDSC. The inadequate function of the host immune system through the down regulation of APC, NK and T cell effectors as well as the elaboration of effector molecules is one of the major mechanisms of tumor immune escape. Similar to increases of MDSC in cancer patients, our data demonstrate that 3LL tumor bearing mice had increased frequency of MDSC in the tumor, blood, spleen and BM as a function of tumor growth. These MDSC functionally suppressed T cell proliferation and APC activities *in vitro*.

The central importance of functional APC in the immune response against cancer has been well defined [25]. The study revealed that even highly immunogenic tumors require host APCs for antigen presentation. Thus, host APC, rather than tumor cells, present tumor antigens. This is consistent with a study indicating that CD8+ T-cell responses can be induced *in vivo* by professional APC that present exogenous antigens in a MHC I-restricted manner [26]. This has been referred to as cross-priming or representation and may be critical for effective antitumor responses [27]. However, in tumor-bearing hosts, there is a state of T-cell unresponsiveness [28], [29], [30]. T cell non responsiveness to specific antigens has been shown to be an early event in tumor progression in animal models of cancer and in cancer patients [31]. The cellular mechanisms that lead to the induction of the tolerogenic state are not well understood. The dominant mechanism underlying the development of antigen-specific T-cell unresponsiveness is thought to be through tumor-antigen processing and presentation by APC [32]. Although T-cell tolerance in cancer has been shown to be mediated by host APC, [25], [32] the nature of these APC have not been clear. Recent studies provide evidence that MDSC may represent a population of APC responsible for induction of antigen-specific CD8 T-cell tolerance in cancer [33], [34]. The tumor microenvironment has immune suppressive mediators such as PGE2, TGF-β, IL-10, VEGF, GM-CSF, IL-6, S100A8/A9 and SCF that recruit and/or activate MDSC [35], [36], [37]. Solid tumors contain a significant proportion of MDSC that maintain an immune suppressive network in the tumor microenvironment.

The impact of MDSC depletion on the APC, T and NK effector activities was evaluated to determine if antitumor innate and adaptive T cell activities could be restored in lung cancer. Broad depletion of MDSC by antibodies that targeted the Gr1 or Ly6G led to decreased MDSC population in the tumor and systemically in the blood, spleen and BM. The APC activity in the tumor was reduced as the tumors progressed, however, MDSC depletion led to increased APC activity in the tumor. Consistent with an increase in APC activity with MDSC depletion, there were increased frequency and activity of NK and T cell effectors in the tumor in comparison to controls. The increased activity of NK cell effectors is significant since these innate immune cells are the first line of defense against tumors and inhibit tumor growth in a non-MHC restricted manner and without prior sensitization to an antigen [38], [39]. CTL and NK cells possess similar cytolytic mechanisms including secretion of perforin and granzyme. Our data indicates that the activated NK and T cell effectors had increased expression of the cytolytic markers granzyme and perforin as well as an increase in IFNγ but reduced IL-10. The cytokine signature of increased IFNγ and reduced IL-10 in the tumor further facilitates antitumor activity. While IFNγ induces anti-angiogenesis through the induction of CXCL9 and CXCL10 [20], reduction in IL-10 can improve APC activity and promote Type I responses [40]. This data indicates that while 3LL tumor growth increased MDSC and reduced APC, NK, and T cell activities, MDSC depletion released the brakes on the functional activities of APC, NK and T cell effectors. In accord with the increased NK and T cell activities, there was an increase in the frequency of apoptotic tumor cells and a concomitant inhibition in tumor burden and migration of tumor cells from the primary tumor site to the lung. The reduction in tumor growth and abrogation in tumor cell migration may be explained by an increase in the frequency of activated T and/or NK effector mediated tumor apoptosis and/or T or NK IFNγ mediated anti-angiogenesis. Based on our observations on increased T and NK cell IFNγ production in the tumor, we evaluated the angiogenic profiles in the tumor. The anti-angiogenic (CXCL9, CXCL10) and pro-angiogenic markers (VEGFa, CXCL2, CXCL5, Angiopoietin 1 and Angiopoietin 2) were quantified in the tumor by RTQPCR. Following MDSC depletion, the anti-angiogenic chemokines CXCL9 and CXCL10 were increased but the pro-angiogenic cytokines VEGFa, Angiopoietin1, Angiopoietin2, CXCL2 and CXCL5 were markedly reduced. Accompanying this profile was a reduction in the endothelial markers MECA 32 in the tumor but an increase in CXCR3 expression. This data indicates that following MDSC depletion there is an influx of IFNγ producing activated T and NK cells that promote angiostasis in the tumor microenvironment by altering the balance of pro and anti angiogenic chemokines. This further suggests that MDSC depletion not only improves APC, NK and T cell immune activities but promotes anti angiogenesis in the tumor that is more effective at controlling tumor growth.

Based on increases in the APC, NK and T cell activities following MDSC depletion, therapeutic vaccination responses were evaluated in *vivo*. In comparison to controls, therapeutic vaccination with BMA-OVA led to decreased tumor burden without complete eradication of the tumors. However following the depletion of MDSC with anti-Gr1 in combination with BMA-OVA vaccine, treated mice had the most substantial reduction in tumor burden with 50% of the mice completely eradicating the tumors and the remainder of mice with a 20 fold reduction in tumor burden in comparison to control. The data indicates that therapeutic vaccination is more effective when combined with MDSC depletion. Long term immunological memory was induced in these mice that were efficient in rejecting a secondary tumor challenge. Flow cytometric evaluation of spleens of mice that had rejected a secondary tumor rechallenge showed an enhancement in the CD4+CD44+ and CD8+CD44+ T cell memory markers as well as an enhanced secretion of IFNγ by memory T cells in response to OVA stimulation *in vitro*. Our findings demonstrate that to engage a coordinated and effective attack against tumors, multiple components of the immune system need to evolve in parallel that require mechanisms leading to immune cell activation with the coordinated disruption of the regulatory mechanisms that limit antitumor immune responses.

Taken together, our data indicate that MDSC depletion reprograms the tumor niche by altering the inflammatory infiltrates in the tumor microenvironment making it permissive for immune destruction of tumors. The benefit of MDSC depletion is further augmented when combined with therapeutic vaccination leading to tumor eradication and immunological memory. Targeting MDSC can improve antitumor immune responses suggesting a broad applicability of combined immune based approaches against a wide range of solid malignancies. This multifaceted approach may prove useful for cancer therapeutics against solid tumors where MDSC play a major role in tumor immune evasion. These results are encouraging and warrant further evaluation of combined MDSC targeting with vaccination approaches for the full therapeutic potential of this strategy in lung cancer and other malignancies.
