# Correction: Myeloid Suppressor Cell Depletion Augments Antitumor Activity in Lung Cancer

**DOI:** 10.1371/annotation/5c756e7d-6e97-416f-836a-dced97cf46af

**Published:** 2012-10-09

**Authors:** Minu K. Srivastava, Li Zhu, Marni Harris-White, Upendra K Kar, Min Huang, Ming F. Johnson, Jay M. Lee, David Elashoff, Robert Strieter, Steven Dubinett, Sherven Sharma

The fourth author's name was spelled incorrectly. The correct name is: Upendra K. Kar.

The correct citation is: Srivastava MK, Zhu L, Harris-White M, Kar UK, Huang M, et al. (2012) Myeloid Suppressor Cell Depletion Augments Antitumor Activity in Lung Cancer. PLoS ONE 7(7): e40677. doi:10.1371/journal.pone.0040677 

